# Open collaborative writing with Manubot

**DOI:** 10.1371/journal.pcbi.1007128

**Published:** 2019-06-24

**Authors:** Daniel S. Himmelstein, Vincent Rubinetti, David R. Slochower, Dongbo Hu, Venkat S. Malladi, Casey S. Greene, Anthony Gitter

**Affiliations:** 1 Department of Systems Pharmacology and Translational Therapeutics, University of Pennsylvania, Philadelphia, Pennsylvania, United States of America; 2 Skaggs School of Pharmacy and Pharmaceutical Sciences, University of California, San Diego, San Diego, California, United States of America; 3 Department of Bioinformatics, University of Texas Southwestern Medical Center, Dallas, Texas, United States of America; 4 Bioinformatics Core Facility, University of Texas Southwestern Medical Center, Dallas, Texas, United States of America; 5 Department of Biostatistics and Medical Informatics, University of Wisconsin-Madison, Madison, Wisconsin, United States of America; 6 Morgridge Institute for Research, Madison, Wisconsin, United States of America; Hebrew University of Jerusalem, ISRAEL

## Abstract

Open, collaborative research is a powerful paradigm that can immensely strengthen the scientific process by integrating broad and diverse expertise. However, traditional research and multi-author writing processes break down at scale. We present new software named Manubot, available at https://manubot.org, to address the challenges of open scholarly writing. Manubot adopts the contribution workflow used by many large-scale open source software projects to enable collaborative authoring of scholarly manuscripts. With Manubot, manuscripts are written in Markdown and stored in a Git repository to precisely track changes over time. By hosting manuscript repositories publicly, such as on GitHub, multiple authors can simultaneously propose and review changes. A cloud service automatically evaluates proposed changes to catch errors. Publication with Manubot is continuous: When a manuscript’s source changes, the rendered outputs are rebuilt and republished to a web page. Manubot automates bibliographic tasks by implementing citation by identifier, where users cite persistent identifiers (e.g. DOIs, PubMed IDs, ISBNs, URLs), whose metadata is then retrieved and converted to a user-specified style. Manubot modernizes publishing to align with the ideals of open science by making it transparent, reproducible, immediate, versioned, collaborative, and free of charge.

## Author summary

Traditionally, scholarly manuscripts have been written in private by a predefined team of collaborators. But now the internet enables realtime open science, where project communication occurs online in a public venue and anyone is able to contribute. Dispersed teams of online contributors require new tools to jointly prepare manuscripts. Existing tools fail to scale beyond tens of authors and struggle to support iterative refinement of proposed changes. Therefore, we created a system called Manubot for writing manuscripts based on collaborative version control. Manubot adopts the workflow from open source software development, which has enabled hundreds of contributors to simultaneously develop complex codebases such as Python and Linux, and applies it to open collaborative writing. Manubot also addresses other shortcomings of current publishing tools. Specifically, all changes to a manuscript are tracked, enabling transparency and better attribution of credit. Manubot automates many tasks, including creating the bibliography and deploying the manuscript as a webpage. Manubot webpages preserve old versions and provide a simple yet interactive interface for reading. As such, Manubot is a suitable foundation for next-generation preprints. Manuscript readers have ample opportunity to not only provide public peer review but also to contribute improvements, before and after journal publication.

This is a *PLOS Computational Biology* Software paper.

## Introduction

The internet enables science to be shared in real-time at a low cost to a global audience. This development has decreased the barriers to making science open, while supporting new massively collaborative models of research [1]. However, the scientific community requires tools whose workflows encourage openness [2]. Manuscripts are the cornerstone of scholarly communication, but drafting and publishing manuscripts has traditionally relied on proprietary or offline tools that do not support *open scholarly writing*, in which anyone is able to contribute and the contribution history is preserved and public. We introduce Manubot, a new tool and infrastructure for authoring scholarly manuscripts in the open, and report how it was instrumental for the collaborative project that led to its creation.

Based on our experience leading a recent open review [3], we discuss the advantages and challenges of open collaborative writing, a form of crowdsourcing [4]. Our review manuscript [5] was code-named the Deep Review and surveyed deep learning’s role in biology and precision medicine, a research area undergoing explosive growth. We initiated the Deep Review in August 2016 by creating a GitHub repository (https://github.com/greenelab/deep-review) to coordinate and manage contributions. GitHub is a platform designed for collaborative software development that is adaptable for collaborative writing. From the start, we made the GitHub repository public under a Creative Commons Attribution License (CC BY 4.0 at https://github.com/greenelab/deep-review/blob/master/LICENSE.md). We encouraged anyone interested to contribute by proposing changes or additions. Although we invited some specific experts to participate, most authors discovered the manuscript organically through conferences or social media, deciding to contribute without solicitation. In total, the Deep Review attracted 36 authors, who were not determined in advance, from 20 different institutions in less than two years.

The Deep Review and other studies that subsequently adopted the Manubot platform were unequivocal successes bolstered by the collaborative approach. However, inviting wide authorship brought many technical and social challenges such as how to fairly distribute credit, coordinate the scientific content, and collaboratively manage extensive reference lists. The manuscript writing process we developed using the Markdown language, the GitHub platform, and our new Manubot tool for automating manuscript generation addresses these challenges.

Manubot supports citations by adding a persistent identifier like a Digital Object Identifier (DOI) or PubMed Identifier (PMID) directly in the text so that large groups of authors do not have to coordinate reference lists. When text is changed, Manubot automatically updates the manuscript’s web page so that all authors can read and edit from the latest version. Because manuscripts are created from GitHub repositories, Manubot supports a workflow where all edits are reviewed and discussed, ensuring that the collaborative text has a cohesive style and message and that authors receive precise credit for their work. These and other features support an open collaborative writing process that is not feasible with other writing platforms.

### Collaborative writing platforms

There are many existing collaborative writing platforms (Table 1) [6]. In general, platforms with “what you see is what you get” (WYSIWYG) editors, such as Microsoft Word or Google Docs, require the least technical expertise to use. On the flip side, WYSIWYG platforms can be difficult to customize and incorporate into automated computational workflows. Traditionally, LaTeX has been used for these needs, since documents are written in plain text and the system is open source and extensible. Rendering LaTeX documents requires specialized software, but webapps like Overleaf now enable collaborative authoring of LaTeX documents. Nonetheless, LaTeX-based systems are limited in that PDF (or similar) is the only fully supported output format. Alternatively, Authorea is a collaborative writing webapp whose primary output format is HTML. Authorea allows authors to write in Markdown, a limited subset of LaTeX, or their WYSIWYG HTML editor.

**Table 1:** Collaborative writing platforms. A summary of features that differentiate Manubot from existing collaborative writing platforms. We assessed features in June 2018 using the free version of each platform and updated our assessment in April 2019 to add the features in the bottom three rows and re-evaluate Authorea and Overleaf. Some platforms offer additional features through a paid subscription or software. 1) Additional functionality, such as bibliography management and tracking changes, is available by editing the Word document stored in OneDrive with the paid Word desktop application. 2) Conversations about modifications take place on the document as comments, annotations, or unsaved chats. There is no integrated forum for discussing and editing revisions. 3) In some circumstances, Overleaf Git commits are not modular. Edits made by distinct authors may be attributed to a single author. The GitHub Sync feature attributes all edits to the project owner.

Feature	Manubot	Authorea	Overleaf v2	Google Docs + Paperpile	Word Online^1^	Markdown on GitHub
Multi-author editing	Yes	Yes	Yes	Yes	Yes	Yes
Propose changes	Yes	No	No	Yes	No	Yes
Continuous integration testing	Yes	No	No	No	No	No
Multi-participant conversation for changes	Yes	No^2^	No^2^	No^2^	No^2^	Yes
Character-level provenance for text	Yes	Yes	No^3^	Requires manual inspection of history	Not after changes are accepted	Yes
Bibliography management	Yes	Yes	Yes	Yes	No, requires the Word desktop application	No
Citation by identifier	Yes	Yes	No	No	No	No
Editing software	Any text editor	Web interface	Web interface	Web interface	Web interface	Any text editor
Document format	Markdown	HTML	LaTeX	Proprietary	Proprietary	Markdown
Templating	Yes	Yes	Yes	No	No	No
Technical expertise required	Yes	No	Yes	No	No	Yes
WYSIWYG mode	No	Yes	Rich text available	Yes	Yes	Preview rendered Markdown
Inline comments	Yes using Hypothesis	Yes	Yes	Yes	Yes	No
Viewing changes	Diff of manuscript source	Highlight changes	Compare labeled versions	Highlight changes	No	Diff of manuscript source

Existing platforms work well for editing text and are widely used for scholarly writing. However, they often lack features that are important for open collaborative writing, such as versatile version control and multiple permission levels. For example, Manubot is the only platform listed in Table 1 that offers the ability to address thematically related changes together and enables multiple authors to iteratively refine proposed changes.

### Manubot contribution workflow

Manubot’s collaborative writing workflow adopts standard software development strategies that enable any contributor to edit any part of the manuscript but enforce discussion and review of all proposed changes. The GitHub platform supports organizing and editing the manuscript. Manubot projects use GitHub *issues* for organization, opening a new issue for each discussion topic. For example, in a review manuscript like the Deep Review, this includes each primary paper under consideration. Within a paper’s issue, contributors summarize the research, discuss it (sometimes with participation from the original authors), and assess its relevance to the review. In a primary research article, issues can instead track progress on specific figures or subsections of text being drafted. Issues serve as an open to-do list and a forum for debating the main messages of the manuscript.

**Fig 1:**
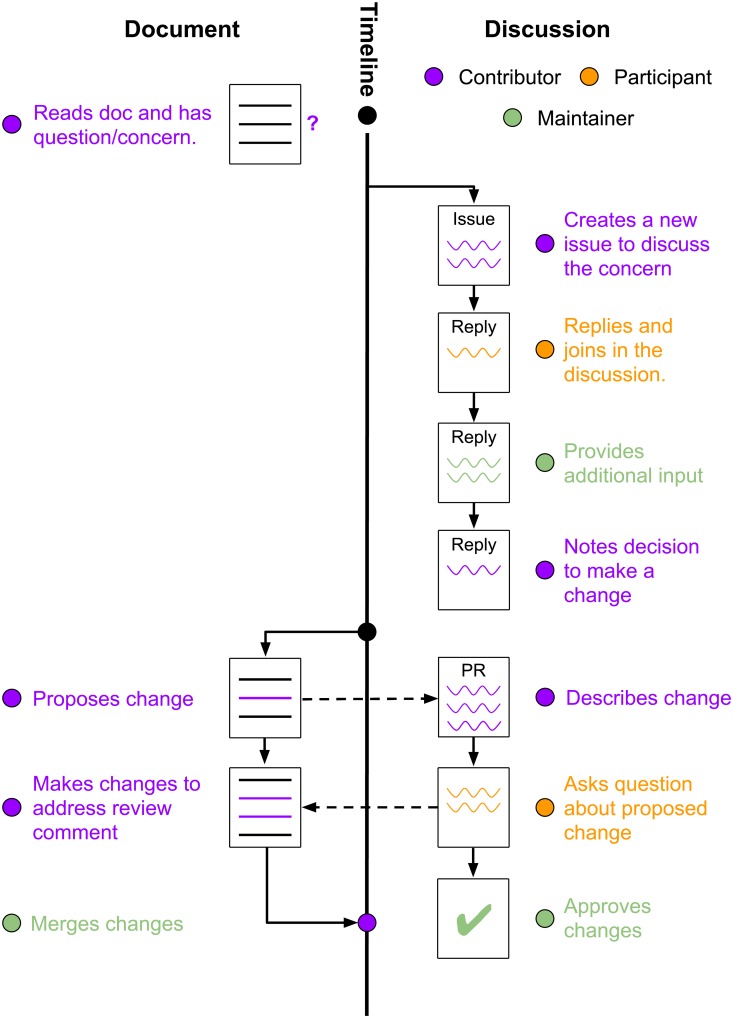
Manubot editing workflow. Any reader can contribute to a Manubot manuscript by proposing a change through a pull request. This example involves three people: a manuscript Maintainer, an existing project Contributor, and an additional Participant in the discussion. Manuscript text is shown in solid lines on the left of the timeline and discussion on GitHub is shown by squiggly lines to the right of the timeline. The Contributor opens a GitHub issue to discuss a manuscript modification. The Maintainer and the Participant provide feedback in the issue, and the Maintainer recommends creating a GitHub pull request to update the text. The Contributor creates the pull request. It is reviewed by the Maintainer and the Participant, and the Contributor updates the pull request in response. Once the pull request is approved, the Maintainer merges the changes into the official version of the manuscript.

GitHub and the underlying Git version control system [7,8] also structure the writing process. The official version of the manuscript is *forked* by individual contributors, creating a copy they can freely modify. A contributor then adds and revises files, grouping these changes into *commits*. When the changes are ready to be reviewed, the series of commits are submitted as a *pull request* through GitHub, which notifies other authors of the pending changes. GitHub’s review interface allows anyone to comment on the changes, globally or at specific lines, asking questions or requesting modifications [9]. Conversations during review can reference other pull requests, issues, or authors, linking the relevant people and content (Fig 1). Reviewing batches of revisions that focus on a single theme is more efficient than independently discussing isolated comments and edits and helps maintain consistent content and tone across different authors and reviewers. Once all requested modifications are made, the manuscript maintainers, a subset of authors with elevated GitHub permissions, formally approve the pull request and merge the changes into the official version. The process of writing and revising material can be orchestrated through GitHub with a web browser (as shown in S1 Video) or through a local text editor.

The Deep Review issue (https://github.com/greenelab/deep-review/issues/575) and pull request (https://github.com/greenelab/deep-review/pull/638) on protein-protein interactions demonstrate this process in practice. A new contributor identified a relevant research topic that was missing from the review manuscript with examples of how the literature would be summarized, critiqued, and integrated into the review. A maintainer confirmed that this was a desirable topic and referred to related open issues. The contributor made the pull request, and two maintainers and another participant made recommendations. After four rounds of reviews and pull request edits, a maintainer merged the changes.

**Fig 2:**
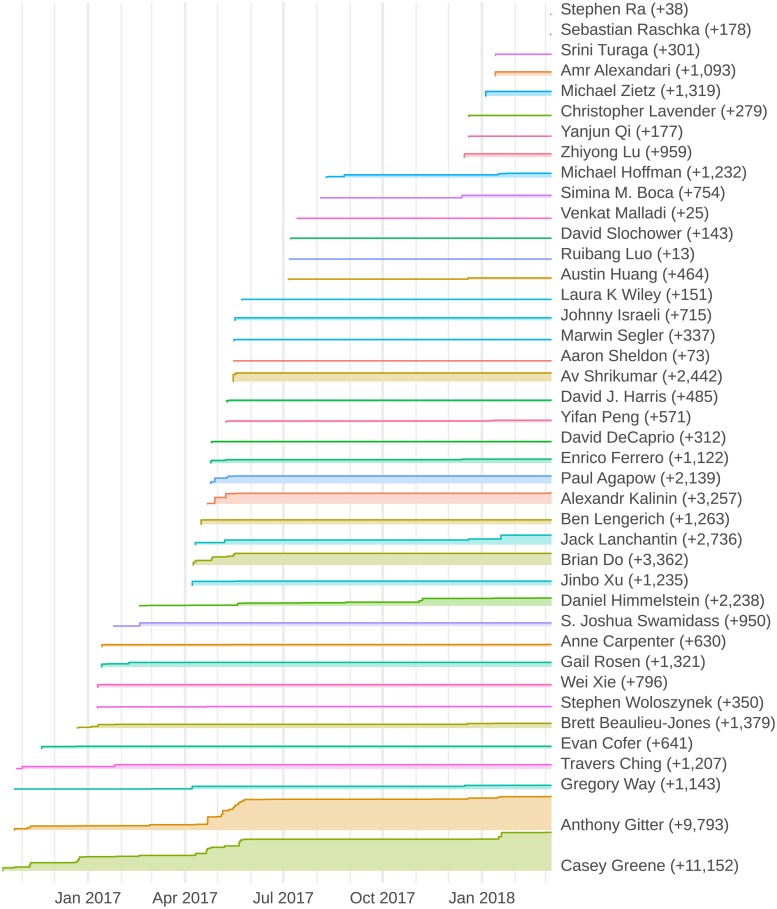
Deep Review contributions by author over time. The total words added to the Deep Review by each author is plotted over time (final values in parentheses). These statistics were extracted from Git commit diffs of the manuscript’s Markdown source. This figure reveals the composition of written contributions to the manuscript at every point in its history. The Deep Review was initiated in August 2016, and the first complete manuscript was released as a preprint [10] in May 2017. While the article was under review, we continued to maintain the project and accepted new contributions. The preprint was updated in January 2018, and the article was accepted by the journal in March 2018 [5]. As of March 06, 2019, the Deep Review repository accumulated 755 Git commits, 317 merged pull requests, 609 issues, and 819 GitHub stars. The notebook to generate this figure can be interactively launched (https://mybinder.org/v2/gh/greenelab/meta-review/binder?filepath=analyses/deep-review-contrib/02.contrib-viz.ipynb) using Binder [11], enabling users to explore alternative visualizations or analyses of the source data.

We found that this workflow was an effective compromise between fully unrestricted editing and a more heavily-structured approach that limited the authors or the sections they could edit. In addition, authors are associated with their commits, which makes it easy for contributors to receive credit for their work. Fig 2 and the GitHub contributors page (https://github.com/greenelab/deep-review/graphs/contributors) summarize all edits and commits from each author, providing aggregated information that is not available on most other collaborative writing platforms. Because the Manubot writing process tracks the complete history through Git commits, it enables detailed retrospective contribution analysis. These pull request and contribution tracking examples both come from Deep Review, the largest Manubot project to date, but illustrate the general principles of transparency and collaboration that are shared by all open Manubot manuscripts.

GitHub issues can also be used for formal peer review by independent or journal-selected reviewers. A reviewer conducting open peer review can create issues using their own GitHub account, as one reviewer did for this manuscript (https://github.com/greenelab/meta-review/issues/124). Alternatively, a reviewer can post feedback with a pseudonymous GitHub account or have a trusted third party such as a journal editor post their comments anonymously. Authors can elect to respond to reviews in the GitHub issues or a public response letter (https://github.com/greenelab/meta-review/blob/v3.0/content/response-to-reviewers.md), creating open peer review.

Although we developed Manubot with collaborative writing in mind, it can also be helpful for individuals preparing scholarly documents. Authors may choose to make their changes directly to the master branch, forgoing pull requests and reviews. This workflow retains many of Manubot’s benefits, such as transparent history, automation, and allowing outside contributors to propose changes. In cases where outside contributions are unwanted, authors can disable pull requests on GitHub. It is also possible to use Manubot on a private GitHub repository. Private manuscripts require some additional customization to disable GitHub Pages and may require a paid continuous integration plan. See the existing manuscripts for examples of the range of contribution workflows and Manubot use cases.

### Manubot features

Manubot is a system for writing scholarly manuscripts via GitHub. For each manuscript, there is a corresponding Git repository. The master branch of the repository contains all of the necessary inputs to build the manuscript. Specifically, a content directory contains one or more Markdown files that define the body of the manuscript as well as a metadata file to set information such as the title, authors, keywords, and language. Figures can be hosted in the content/images subdirectory or elsewhere and specified by URL. Repositories contain scripts and other files that define how to build and deploy the manuscript. Many of these operations are delegated to the manubot Python package or other dependencies such as Pandoc, which converts between document formats, and Travis CI, which builds the manuscript in the cloud. Manubot pieces together many existing standards and technologies to encapsulate a manuscript in a repository and automatically generate outputs.

#### Markdown

With Manubot, manuscripts are written as plain-text Markdown files. The Markdown standard itself provides limited yet crucial formatting syntax, including the ability to embed images and format text via bold, italics, hyperlinks, headers, inline code, codeblocks, blockquotes, and numbered or bulleted lists. In addition, Manubot relies on extensions from Pandoc Markdown to enable citations, tables, captions, and equations specified using the popular TeX math syntax. Markdown with Pandoc extensions supports most formatting options required for scholarly writing [12] but currently lacks the ability to cross-reference and automatically number figures, tables, and equations. For this functionality, Manubot includes the pandoc-xnos suite of Pandoc filters. A list of formatting options officially supported by Manubot, at the time of writing, is viewable as raw Markdown (https://github.com/manubot/rootstock/raw/091ca8d85c8ef2d7af16fcc8d2ed3ebcbc187f13/content/02.delete-me.md) and the corresponding rendered HTML (https://manubot.github.io/rootstock/v/091ca8d85c8ef2d7af16fcc8d2ed3ebcbc187f13/).

By virtue of its readable syntax, Markdown is well suited for version control using Git. Markdown treats a single line break between text as a space and requires two-or-more consecutive line breaks to denote a new paragraph. For optimal tracking of Markdown files with Git, we recommend placing each sentence on its own line. This convention allows Git to display diffs on a per sentence basis, avoids unnecessary reflows associated with line wrapping, and supports easy rearrangement of sentences.

#### Citation by identifier

Manubot includes an additional layer of citation processing, currently unique to the system. All citations point to a standard identifier, for which Manubot automatically retrieves bibliographic metadata such as the title, authors, and publication date. Table 2 presents the supported identifiers and example citations before and after Manubot processing. Authors can optionally define citation tags to provide short readable alternatives to the citation identifiers. Citation metadata is exported to the Citation Style Language (CSL) JSON Data Items format, an open standard that is widely supported by reference managers [13,14]. However, sometimes external resources provide Manubot with invalid CSL Data, which can cause errors with downstream citation processors, such as pandoc-citeproc (http://hackage.haskell.org/package/pandoc-citeproc). Therefore, Manubot removes invalid fields according to the CSL Data specification (https://github.com/citation-style-language/schema). In cases where automatic retrieval of metadata fails or produces incorrect references—which is most common for URL citations—users can manually provide the correct metadata using common reference formats. Manual metadata also supports references without standard identifiers, such as print-only newspaper articles.

**Table 2:** Citation types supported by Manubot. Manubot allows users to cite different types of persistent identifiers. Metadata source indicates the primary resource used to retrieve bibliographic metadata. For certain identifier types, additional metadata sources are queried should the primary fail. For example, when translation-server ISBN lookup fails, Manubot tries Wikipedia’s Citoid (https://www.mediawiki.org/wiki/Citoid) service followed by the isbnlib (https://github.com/xlcnd/isbnlib) Python package. When translation-server URL lookup fails, Manubot then tries Greycite (http://greycite.knowledgeblog.org/) [15]. Raw citations enable citing works when no supported persistent identifiers exist, but require that the user specifies the metadata. Finally, authors may optionally map a named tag to any of the supported identifier types. In this example, the tag avasthi-preprints represents the DOI identifier 10.7554/eLife.38532. API: application programming interface.

Identifier	Metadata source	Example citation	Processed citation
Digital Object Identifier (DOI)	DOI Content Negotiation	doi:10.1098/rsif.2017.0387	[5]
shortDOI	DOI Proxy Server API	doi:10/gddkhn	[5]
PubMed Identifier (PMID)	NCBI E-utilities	pmid:25851694	[16]
PubMed Central Identifier (PMCID)	NCBI Literature Citation Exporter	pmcid:PMC4719068	[4]
arXiv ID	arXiv API	arxiv:1502.04015v1	[17]
International Standard Book Number (ISBN)	Zotero translation-server	isbn:9780262517638	[18]
Web address (URL)	Zotero translation-server	url:https://lgatto.github.io/open-and-open/	[19]
Wikidata ID	Zotero translation-server	wikidata:Q56458321	[20]
Raw	Provided by user	raw:paywall-movie	[21]
Tag	Source for tagged identifier	tag:avasthi-preprints	[22]

Manubot formats bibliographies according to a CSL style specification. Styles define how references are constructed from bibliographic metadata, controlling layout details such as the maximum number of authors to list per reference. Manubot’s default style emphasizes titles and electronic (rather than print) identifiers and applies numeric-style citations [23]. Alternatively, users can also choose from thousands of predefined styles (http://editor.citationstyles.org/searchByName/) or build their own [24]. As a result, adopting the specific bibliographic format required by a journal usually just requires specifying the style’s source URL in the Manubot configuration.

#### Format conversion

Manubot uses Pandoc (https://pandoc.org/) to convert manuscripts from Markdown to HTML, PDF, and optionally DOCX outputs. Pandoc also supports Journal Article Tag Suite (JATS), a standard format for scholarly articles that is used by publishers, archives, and text miners [25–27]. Pandoc’s JATS support provides an avenue to integrate Manubot with the larger JATS ecosystem. In the future, journals may accept submissions in JATS. For now, Manubot’s DOCX output is usually sufficient for journal submissions that require an editable source document. Otherwise, authors generally use the PDF output for preprint and initial journal submissions. The primary Manubot output is HTML intended to be viewed in a web browser. Accordingly, manuscripts natively support JavaScript and can thus include any web-based interactive visualization, such as those produced using Vega-Lite (https://vega.github.io/vega-lite/), Bokeh (https://bokeh.pydata.org/), or Plotly (https://plot.ly/) [28,29].

#### Interactive features and appearance

**Fig 3:**
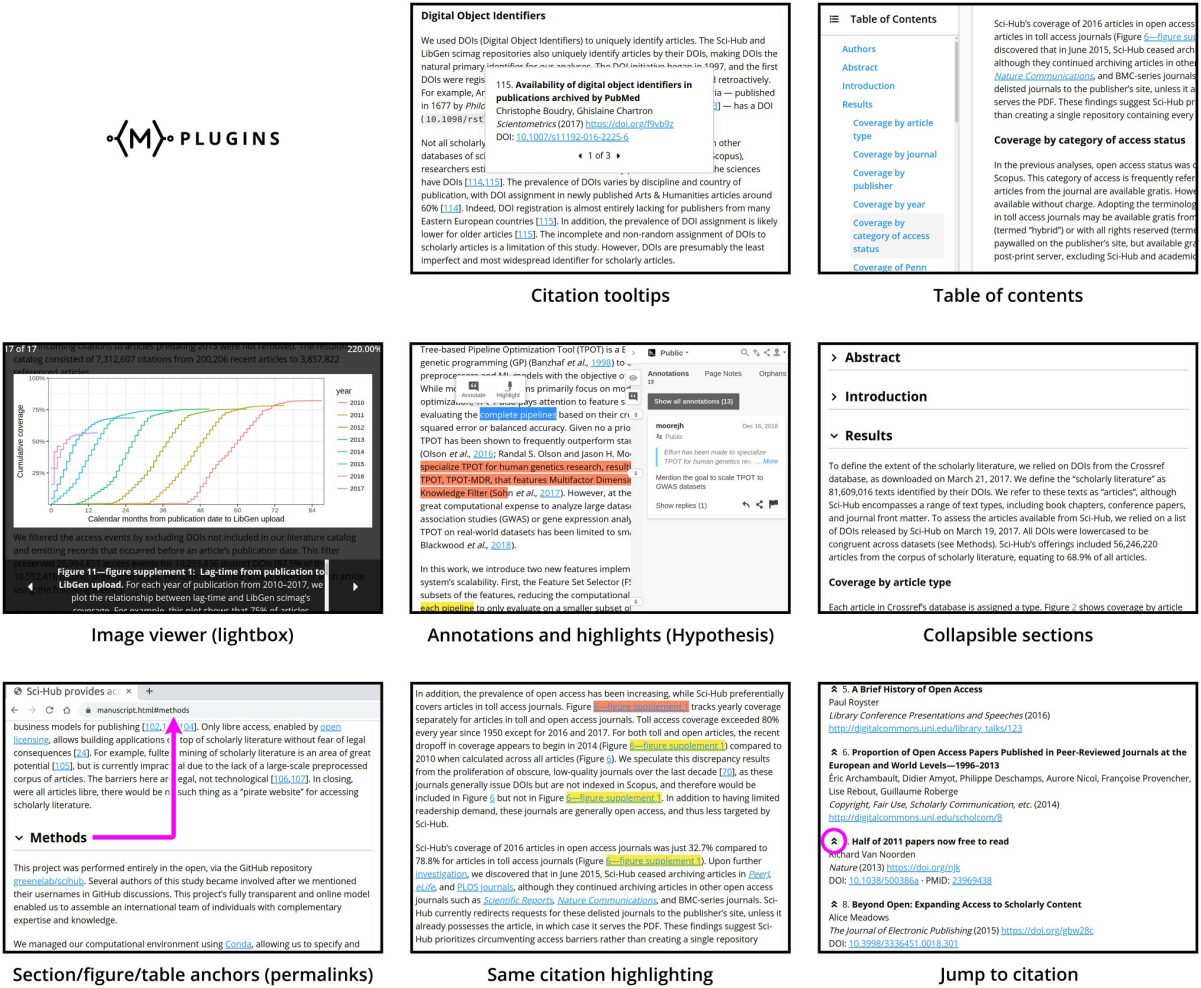
Examples of the various Manubot plugins, illustrating their functionality and usefulness. Screenshots were taken from existing manuscripts made with Manubot: Sci-Hub Coverage Study (https://greenelab.github.io/scihub-manuscript/v/fd7acb7ed0108c920da56f84819ce13f02f68aa8/) and TPOT-FSS (https://trang1618.github.io/tpot-fss-ms/), available under the CC BY 4.0 License. Clarifying markups are overlaid in purple.

Manubot comes with several “plugins” that can be included in manuscripts exported as HTML. These plugins add special interactive features that enhance the user experience of viewing and reading manuscripts (Fig 3). For example, with the “tooltips” plugin enabled, when the user hovers over a link to a reference or figure, a preview of that item pops up above the link, along with controls to navigate between other mentions of that item elsewhere in the document. The build process can also accommodate different “themes”, which change the general aesthetics and appearance of the exported document (e.g. from a contemporary sans-serif style to a more traditional serif style). The architecture of the plugins and themes is designed to provide authors with enough flexibility to suit their particular needs and preferences.

The Manubot “front-end” (layout, look, controls, behavior, etc.) was developed in line with current best practices and user expectations of the modern web. The plugins use standard technology built in to most major web browsers, allowing them to be relatively lightweight, modular, and easy to configure.

#### Continuous publication

Manubot performs continuous publication: Every update to a manuscript’s source is automatically reflected in the online outputs. The approach uses continuous integration (CI) [30–32], specifically via Travis CI, to monitor changes. When changes occur, the CI service attempts to generate an updated manuscript. If this process is error free, the CI service timestamps the manuscript and uploads the output files to the GitHub repository. Because the HTML manuscript is hosted using GitHub Pages, the CI service automatically deploys the new manuscript version when it pushes the updated outputs to GitHub. Using CI to build the manuscript automatically catches many common errors, such as misspelled citations, invalid formatting, or misconfigured software dependencies.

To illustrate, the source GitHub repository for this article is https://github.com/greenelab/meta-review. When this repository changes, Travis CI rebuilds the manuscript (https://travis-ci.org/greenelab/meta-review). If successful, the output is deployed back to GitHub (to dedicated output and gh-pages branches). As a result, https://greenelab.github.io/meta-review stays up to date with the latest HTML manuscript. Furthermore, versioned URLs, such as https://greenelab.github.io/meta-review/v/4b6396bcefd1b9c7ddf39c1d3f0b3eab2dd63f31/, provide access to previous manuscript versions.

#### Timestamping

The idea of the “priority of discovery” is important to science, and Vale and Hyman discuss the importance of both disclosure and validation [33]. In their framework, disclosure occurs when a scientific output is released to the world. However, for a manuscript that is shared as it is written, being able to establish priority could be challenging. Manubot supports OpenTimestamps (https://opentimestamps.org/) to timestamp the HTML and PDF outputs on the Bitcoin blockchain. This procedure allows one to retrospectively prove that a manuscript version existed prior to its blockchain-verifiable timestamp [17,34–37]. Timestamps protect against attempts to rewrite a manuscript’s history and ensure accurate histories, potentially alleviating certain authorship or priority disputes. Because all Bitcoin transactions compete for limited space on the blockchain, the fees required to send a single transaction can be high. OpenTimestamps minimizes fees by encoding many timestamps into a single Bitcoin transaction, enabling the service to be free of charge [38]. Since transactions can take up to a few days to be made, Manubot initially stores incomplete timestamps and upgrades them in future continuous deployment builds. We find that this asynchronous design with timestamps precise to the day is suitable for the purposes of scientific writing.

#### Reproducible manuscripts

Manubot and its dependencies are free of charge and largely open source. It does rely on gratis services from two proprietary platforms: GitHub and Travis CI. Fortunately, lock-in to these services is minimal, and several substitutes already exist. Manubot provides a substantial step towards end-to-end document reproducibility, where every figure or piece of data in a manuscript can be traced back to its origin [39] and is well-suited for preserving provenance. For example, figures can be specified using versioned URLs that refer to the code that created them. In addition, manuscripts can be templated, so that numerical values or tables are inserted directly from the repository that created them. The Fig 2 caption provides examples of templates. Phrases such as “755 Git commits” are written as {{total_commits}} Git commits so that the commit count can be automatically updated.

#### Getting started

An example repository at https://github.com/manubot/rootstock, referred to as Rootstock, demonstrates Manubot’s features and serves as a template for users to write their own manuscripts with Manubot. The current setup process includes cloning the Rootstock repository, rebranding it to the user’s manuscript, and configuring continuous integration. The setup process is complex but must only be performed once per manuscript. Incorporating new Manubot features into an existing manuscript is also possible by pulling the latest commits from Rootstock, which sometimes involves resolving Git conflicts.

Contributing to a manuscript is less technical and can be performed entirely through GitHub’s web interface, as discussed in the contribution workflow section and demonstrated in S1 Video. Interested readers can practice editing a demo manuscript at https://github.com/manubot/try-manubot.

At the 2019 *Pacific Symposium on Biocomputing*, we led a working group where 17 conference participants contributed to a different demo manuscript (https://git.dhimmel.com/psb-manuscript/). Based on this experience, we believe most computational scholars have the expertise to contribute to a Manubot manuscript. Proficiency with Manubot requires familiarity with Markdown, Git, GitHub, and continuous integration. While these tools do present a barrier to entry, they are also highly applicable outside of Manubot and increasingly part of the standard curriculum for computational scholars. For example, Markdown is used for documenting Jupyter and R Markdown notebooks.

#### Existing manuscripts

Since its creation to facilitate the Deep Review, Manubot has been used to write a variety of scholarly documents. The Sci-Hub Coverage Study (https://github.com/greenelab/scihub-manuscript)—performed openly on GitHub from its inception—investigated Sci-Hub’s repository of pirated articles [40]. Sci-Hub reviewed (https://github.com/greenelab/scihub-manuscript/issues/17) the initial preprint from this study in a series of tweets, pointing out a major error in one of the analyses. Within hours, the authors used Markdown’s strikethrough formatting in Manubot to cross-out the errant sentences (commit at https://github.com/greenelab/scihub-manuscript/commit/8fcd0cd665f6fb5f39bed7e26b940aa27d4770ba, versioned manuscript) at https://greenelab.github.io/scihub-manuscript/v/8fcd0cd665f6fb5f39bed7e26b940aa27d4770ba/, thereby alerting readers to the mistake and preventing further propagation of misinformation. One month later, a larger set of revisions (https://github.com/greenelab/scihub-manuscript/pull/19) explained the error in more detail and was included in a second version of the preprint. As such, continuous publication via Manubot helped the authors address the error without delay, while retaining a public version history of the process. This Sci-Hub Coverage Study preprint was the most viewed (http://web.archive.org/web/20171221221858/http://www.prepubmed.org/top_preprints/) 2017 *PeerJ Preprint*, while the Deep Review was the most viewed 2017 *bioRxiv* preprint [41]. Hence, in Manubot’s first year, two of the most popular preprints were written using its collaborative, open, and review-driven authoring process.

Additional research studies are being authored using Manubot, spanning the fields of regulatory genomics (https://vsmalladi.github.io/tfsee-manuscript/ and https://simonvh.github.io/gimmemotifs-manuscript/) [42], synthetic biology (https://zach-hensel.github.io/low-noise-manuscript/) [43], climate science (https://openclimatedata.github.io/global-emissions/), visual perception (https://laurentperrinet.github.io/2019-05_illusions-visuelles/) [44], machine learning (https://trang1618.github.io/tpot-fss-ms/) [45], computational toolkits (https://jmonlong.github.io/manu-vgsv/) [46], and data visualization (https://yt-project.github.io/yt-3.0-paper/). Manubot is also being used for documents beyond traditional journal publications, such as research tips (https://benjamin-lee.github.io/deep-rules/), quality standards (https://indigo-dc.github.io/sqa-baseline/) [47], grant proposals (https://greenelab.github.io/manufund-2018/), progress reports (https://greenelab.github.io/czi-hca-report/), undergraduate research reports (https://zietzm.github.io/Vagelos2017/) [48], literature reviews (https://slochower.github.io/synthetic-motor-literature/), and lab notebooks. Finally, manuscripts written with other authoring systems have been successfully ported to Manubot, including the Bitcoin Whitepaper (https://git.dhimmel.com/bitcoin-whitepaper/) [49] and Project Rephetio (https://git.dhimmel.com/rephetio-manuscript/) manuscript [50].

#### Citation utilities

The manubot Python package provides easy access to Manubot’s citation-by-identifier infrastructure, whose functionality extends beyond just Manubot manuscripts. For example, the Kipoi (https://kipoi.org/) model zoo for genomics [51] uses Manubot’s Python interface to retrieve model authors from persistent identifiers. In addition, the manubot cite command line utility takes a list of citations and returns either a rendered bibliography or CSL Data Items (i.e. JSON-formatted reference metadata). For example, the following command outputs a Markdown reference list for the two specified articles according to the bibliographic style of *PeerJ*:

manubot cite --render --format = markdown \

 --csl = https://github.com/citation-style-language/styles/raw/master/peerj.csl \

 pmid:29618526 doi: 10.1038/550143a

Pandoc brands itself as a “universal document converter”, and can convert from any of 32 input formats to any of 51 output formats as of version 2.7. Thanks to its versatility and active development since 2006, Pandoc enjoys a large userbase across many disciplines and applications. Its filter interface enables adding custom functionality with community-developed programs. We are prototyping a Manubot-based citation-by-identifier filter. This filter would allow Pandoc users to cite persistent identifiers as part of their existing Pandoc workflows, without requiring them to adopt other aspects of Manubot. It could help popularize citation-by-identifier at an influential scale.

#### Future enhancements

Manubot is still under active development, and we envision major changes in its design and dependencies going forward. Currently, manuscript repositories must contain a large number of files that do not directly contain manuscript content. While this enables a high-degree of customization, it also increases complexity. Therefore, we are investigating whether configuration files with sensible defaults could enable bare-bones repositories that contain manuscript content and little else.

In addition to simplifying the usage, we’re also looking into simplifying the setup. Presently, setup is complex because users must do advanced command-line operations to clone the Rootstock repository and configure Travis CI. Although we provide detailed instructions, users often struggle to replicate the long list of setup commands in an appropriate computational environment. One priority will be to automate setup to a higher degree. However, this may require switching the services Manubot uses for continuous integration (e.g. from Travis CI to GitHub Actions, CircleCI, Drone, or GitLab CI), environment management (e.g. from Conda to Docker), and repository hosting (e.g. from GitHub to GitLab). In addition to simplifying setup, such migrations may also present the opportunity to decrease dependency on proprietary services and address other Manubot shortcomings, such as the current inability to view rendered manuscripts produced by pull request builds.

Upgrading a Manubot instance is an opt-in procedure. Therefore, when we introduce fundamental changes, existing manuscripts continue to function. However, large Rootstock changes can make upgrading existing manuscripts difficult. We are happy to provide users *pro bono* assistance to upgrade or troubleshoot manuscripts. Users can open an issue (https://github.com/manubot/rootstock/issues) at the Rootstock repository for help.

One strategy to grow Manubot usage is to identify a specific user group or use case for which Manubot can be widely adopted. For example, a journal may decide to build their publishing workflow around Manubot, such that submissions would consist of a Manubot repository. This application would be most suitable for journals that currently use GitHub for submissions and publishing, such as the *Journal of Open Source Software* [52]. Manubot could also gain traction as the primary tool used to write collaborative manuscripts within certain communities. For example, open research projects built from voluntary contributions by geographically-distributed individuals could adopt Manubot. Likewise, Manubot may excel at enabling collaborative translation of existing manuscripts into other languages. Another application could be collaborative development of online lessons, documentation, or tutorials. Projects like Software Carpentry already host each lesson in a separate GitHub repository and may benefit from Manubot-generated permalinks to historical versions.

## Authorship

Manubot does not impose any restrictions on authorship. It allows authors to adhere to the author inclusion and ordering conventions of their field, which vary considerably across disciplines [53]. Some Manubot projects create a table in their GitHub repository to track contributors who did not commit text to the manuscript (https://github.com/Benjamin-Lee/deep-rules/blob/cfb7f744573ca0532a19ca1a8e9473a555cf8eb2/contributors.md). This provides a transparent way to record contributions such as experimental research that generated data for the manuscript and discuss whether they meet that project’s authorship criteria. Contribution transparency helps prevent ghostwriting [54] and is especially important in collaborative writing [55]. Although we recommend authors provide their ORCID and GitHub username, Manubot also supports pseudonyms, pseudonymous GitHub usernames, and authors without an ORCID or GitHub account.

To determine authorship for the Deep Review, we followed the International Committee of Medical Journal Editors (ICMJE) guidelines and used GitHub to track contributions. ICMJE recommends authors substantially contribute to, draft, approve, and agree to be accountable for the manuscript. We acknowledged other contributors who did not meet all four criteria, including contributors who provided text but did not review and approve the complete manuscript. Although these criteria provided a straightforward, equitable way to determine who would be an author, they did not produce a traditionally ordered author list. In biomedical journals, the convention is that the first and last authors made the most substantial contributions to the manuscript. This convention can be difficult to reconcile in a collaborative effort. Using Git, we could quantify the number of commits each author made or the number of sentences an author wrote or edited, but these metrics discount intellectual contributions such as discussing primary literature and reviewing pull requests. Therefore, we concluded that it is not possible to construct an objective system to compare and weight the different types of contributions and produce an ordered author list [56].

To address this issue, we generalized the concept of “co-first” authorship, in which two or more authors are denoted as making equal contributions to a paper. We defined four types of contributions [5], from major to minor, and reviewed the GitHub discussions and commits to assign authors to these categories. A randomized algorithm then arbitrarily ordered authors within each contribution category, and we combined the category-specific author lists to produce a traditional ordering. The randomization procedure was shared with the authors in advance (pre-registered) and run in a deterministic manner. Given the same author contributions, it always produced the same ordered author list. We annotated the author list to indicate that author order was partly randomized and emphasize that the order did not indicate one author contributed more than another from the same category. The Deep Review author ordering procedure illustrates authorship possibilities when all contributions are publicly tracked and recorded that would be difficult with a traditional collaborative writing platform.

Papers with hundreds or thousands of authors are on the rise, such as the article describing the experiments and data that led to the discovery of the Higgs Boson [57] (5000 authors) and the report of the *Drosophila* genome [58] (1000 authors). Yet the number of people that participated in writing those papers, as opposed to generating and analyzing the data, is not always clear and is likely to be far below the number of authors [59,60]. Manubot provides the scientists involved in large collaborations the opportunity to actively participate, through a public forum, in the writing process.

## Discussion

### Collaborative review manuscripts

The open scholarly writing Manubot enables has particular benefits for review articles, which present the state of the art in a scientific field [61]. Literature reviews are typically written in private by an invited team of colleagues. In contrast, broadly opening the process to anyone engaged in the topic—such that planning, organizing, writing, and editing occur collaboratively in a public forum where anyone is welcome to participate—can maximize a review’s value. Open drafting of reviews is especially helpful for capturing state-of-the-art knowledge about rapidly advancing research topics at the intersection of existing disciplines where contributors bring diverse opinions and expertise.

Writing review articles in a public forum allows review authors to engage with the original researchers to clarify their methods and results and present them accurately, as exemplified at https://github.com/greenelab/deep-review/issues/213. Additionally, discussing manuscripts in the open generates valuable pre-publication peer review of preprints [22] or post-publication peer review [16,62,63]. Because incentives to provide public peer review of existing literature [64] are lacking, open collaborative reviews—where authorship is open to anyone who makes a valid contribution—could help spur more post-publication peer review.

### Additional collaborative writing projects

The Deep Review was not the first scholarly manuscript written online via an open collaborative process. In 2013, two dozen mathematicians created the 600-page Homotopy Type Theory book, writing collaboratively in LaTeX on GitHub [65,66]. Two technical books on cryptocurrency—Mastering Bitcoin (https://github.com/bitcoinbook/bitcoinbook) and Mastering Ethereum (https://github.com/ethereumbook/ethereumbook)—written on GitHub in AsciiDoc format have engaged hundreds of contributors. Both Homotopy Type Theory and Mastering Bitcoin continue to be maintained years after their initial publication. A 2017 perspective on the future of peer review was written collaboratively on Overleaf, with contributions from 32 authors [67]. While debate was raging over tightening the default threshold for statistical significance, nearly 150 scientists contributed to a Google Doc discussion that was condensed into a traditional journal commentary [68,69]. The greatest success to date of open collaborative writing is arguably Wikipedia, whose English version contains over 5.5 million articles. Wikipedia scaled encyclopedias far beyond any privately-written alternative. These examples illustrate how open collaborative writing can scale scholarly manuscripts where diverse opinion and expertise are paramount beyond what would otherwise be possible.

Open writing also presents new opportunities for distributing scholarly communication. Though it is still valuable to have versioned drafts of a manuscript with digital identifiers, journal publication may not be the terminal endpoint for collaborative manuscripts. After releasing the first version of the Deep Review [10], 14 new contributors updated the manuscript (Fig 2). Existing authors continue to discuss new literature, creating a living document. Manubot provides an ideal platform for perpetual reviews [70,71].

Concepts for the future of scholarly publishing extend beyond collaborative writing [72–74]. Pandoc Scholar [12] and Bookdown [75], which has been used for open writing [76], both enhance traditional Markdown to better support publishing. The knitcitations (https://github.com/cboettig/knitcitations) package enables citation by DOI or URL in R Markdown documents. Examples of continuous integration to automate manuscript generation include gh-publisher (https://github.com/ewanmellor/gh-publisher) and jekyll-travis (https://github.com/mfenner/jekyll-travis), which was used to produce a continuously published webpage (http://book.openingscience.org/) for the Opening Science book [77,78]. Binder [11], Distill journal articles [79], Idyll [80], and Stencila [81,82] support manuscripts with interactive graphics and close integration with the underlying code. As an open source project, Manubot can be extended to adopt best practices from these other emerging platforms.

Several other open science efforts are GitHub-based like our collaborative writing process. ReScience [83] as well as titles from Open Journals, such as the *Journal of Open Source Software* [52], rely on GitHub for peer review and hosting. Distill uses GitHub for transparent peer review and post-publication peer review [84]. GitHub is increasingly used for resource curation [85], and collaborative scholarly reviews combine literature curation with discussion and interpretation.

### Limitations

There are potential limitations of our GitHub-based approach. Because the Deep Review pertained to a computational topic, most of the authors had computational backgrounds, including previous experience with version control workflows and GitHub. In other disciplines, collaborative writing via GitHub and Manubot could present a steeper barrier to entry and deter participants. In addition, Git carefully tracks all revisions to the manuscript text but not the surrounding conversations that take place through GitHub issues and pull requests. These discussions must be archived to ensure that important decisions about the manuscript are preserved and authors receive credit for intellectual contributions that are not directly reflected in the manuscript’s text. GitHub supports programmatic access to issues, pull requests, and reviews so tracking these conversations is feasible in the future.

In the Deep Review, we established contributor guidelines (https://github.com/greenelab/deep-review/blob/v1.0/CONTRIBUTING.md) that discussed norms in the areas of text contribution, peer review, and authorship, which we identified in advance as potential areas of disagreement. Our contributor guidelines required verifiable participation for authorship: either directly attributable changes to the text or participation in the discussion on GitHub. These guidelines did not discuss broader community norms that may have improved inclusiveness. It is also important to consider how the move to an open contribution model affects under-represented minority members of the scientific community [19]. Recent work has identified clear social norms and processes as helpful to maintaining a collaborative culture [86]. Conferences and open source projects have used codes of conduct to establish these norms (e.g. Contributor Covenant at https://www.contributor-covenant.org/) [87]. We would encourage the maintainers of similar projects to consider broader codes of conduct for project participants that build on social as well as academic norms.

### Manubot in the context of open science

Science is undergoing a transition towards openness. The internet provides a global information commons, where scholarship can be publicly shared at a minimal cost. For example, open access publishing provides an economic model that encourages maximal dissemination and reuse of scholarly articles [18,88,89]. More broadly, open licensing solves legal barriers to content reuse, enabling any type of scholarly output to become part of the commons [90,91]. The opportunity to reuse data and code for new investigations, as well as a push for increased reproducibility, has begot a movement to make all research outputs public, unless there are bona fide privacy or security concerns [92–94]. New tools and services make it increasingly feasible to publicly share the unabridged methods of a study, especially for computational research, which consists solely of software and data.

Greater openness in both research methods and publishing creates an opportunity to redefine peer review and the role journals play in communicating science [67]. At the extreme is real-time open science, whereby studies are performed entirely in the open from their inception [95]. Many such research projects have now been completed, benefiting from the associated early-stage peer review, additional opportunity for online collaboration, and increased visibility [50,96].

Manubot is an ideal authoring protocol for real-time open science, especially for projects that are already using an open source software workflow to manage their research. While Manubot does require technical expertise, the benefits are manyfold. Specifically, Manubot demonstrates a system for publishing that is transparent, reproducible, immediate, permissionless, versioned, automated, collaborative, open, linked, provenanced, decentralized, hackable, interactive, annotated, and free of charge. These attributes empower integrating Manubot with an ecosystem of other community-driven tools to make science as open and collaborative as possible.

## Supporting information

S1 Video Editing a manuscript on GitHub. This screen recording demonstrates how to propose edits to a Manubot manuscript via GitHub. In the video [97], a contributor creates a pull request to add a sentence to the try-manubot manuscript. The contributor then revises the proposed change to add a citation, after which it is accepted, merged, and automatically deployed.
(MP4)
